# Predicting peroxisome proliferator-activated receptor gamma potency of small molecules: a synergistic consensus model and deep learning binding affinity approach powered by Enalos Cloud Platform

**DOI:** 10.1007/s11030-025-11230-6

**Published:** 2025-06-14

**Authors:** Maria Antoniou, Konstantinos D. Papavasileiou, Antreas Tsoumanis, Georgia Melagraki, Antreas Afantitis

**Affiliations:** 1 Department of ChemoInformatics, NovaMechanics Ltd, 1070 Nicosia, Cyprus; 2 Computation-Based Science and Technology Research Centre, The Cyprus Institute, 2121 Nicosia, Cyprus; 3 Entelos Institute, 6059 Larnaca, State Cyprus; 4 Department of ChemoInformatics, NovaMechanics MIKE, 18545 Piraeus, Greece; 5 Division of Physical Sciences Applications, Hellenic Military Academy, 16672 Vari, Greece

**Keywords:** Nuclear receptors, PPAR antagonists, PFAS, Structure–activity relationships, Machine learning, Enalos Cloud Platform

## Abstract

Peroxisome proliferator-activated receptor gamma (PPARγ) antagonists play a critical role in regulating glucose and lipid metabolism, making them promising candidates for antidiabetic therapies. To support the ongoing search of such compounds, this study introduces two advanced in silico models for predicting the binding affinity and biological activity of small molecules targeting PPARγ. A neural network was developed to classify compounds as strong or weak binders based on molecular docking scores. Additionally, a consensus model combining Random Forest, Support Vector Machine, and k-Nearest Neighbours algorithms was implemented to predict the antagonistic activity of small molecules. Both models were rigorously validated according to the Organisation for Economic Co-operation and Development (OECD) guidelines, to ensure generalisability and sufficient efficiency in detecting the minority class (active antagonists). Mechanistic insights into how key molecular descriptors influence PPARγ activity were discussed in a posteriori interpretation. A case study involving 34 prioritised per- and polyfluoroalkyl substances (PFAS) were screened with the developed workflows to demonstrate their practical application. The models, integrated into user-friendly web applications via the Enalos Cloud Platform, enable accessible and efficient virtual screening, supporting the discovery of PPARγ modulators.

**Supplementary Information:**

The online version contains supplementary material available at 10.1007/s11030-025-11230-6.

## Introduction

Peroxisome proliferator-activated receptors (PPARs) are members of the NR1C nuclear receptor (NR) subfamily of ligand-activated transcription factors [1]. They are essential in regulating gene expression by binding to PPAR response elements (PPREs) in the promoter regions of target genes. Upon binding, PPARs form heterodimers with retinoid X receptors (RXRs), which are also members of the nuclear receptor superfamily [2, 3]. This family comprises three main isoforms with structural similarities: PPARα (NR1C1), PPARβ/δ (NR1C2), and PPARγ (NR1C3), each exhibiting unique tissue functions [4]. PPARα is primarily expressed in the liver and promotes the oxidation of fatty acids [5], whilst PPARβ/δ is involved in fatty acid metabolism [6]. PPARγ, the most intensively studied NR1C receptor, plays a vital part in energy homeostasis and metabolic function. Its activation is central to insulin sensitisation, adipogenesis, and the regulation of glucose and lipid metabolism [3, 7, 8].

The search for novel PPAR activators remains a dynamic area of research, with ongoing efforts aiming to discover selective or dual modulators that target PPARγ and δ receptors [9, 10]. Known PPARγ full agonists, such as thiazolidinediones (rosiglitazone and pioglitazone), are used therapeutically for the treatment of type 2 diabetes mellitus, owing to their efficient insulin-sensitising abilities. However, the association of these drugs with adverse side effects, such as weight gain, fluid retention, and bone fractures, has restricted their clinical use [11]. Unlike agonists, which stabilise helix 12 (H12) in its active conformation through hydrogen bonds, antagonists induce a distinct orientation of H12 which closes the entrance to the ligand-binding site [12]. As a result, PPARγ antagonists have emerged as alternatives to full agonists for use as clinically effective antidiabetic therapies [13, 14].

Quantitative Structure–Property/Activity Relationships (QSPRs/QSARs) have long been utilised to assess the biological activity of compounds by establishing statistical correlations between their molecular structure with physicochemical properties or bioactivities [15]. These computational methods are crucial in early-stage drug discovery and chemical risk assessment, since they are cost- and time-effective alternatives to experimental screenings. The fundamental assumption of QSARs/QSPRs is that identical chemical structures are most likely to exhibit similar biological effects. They rely on mathematical representations (i.e., two-dimensional descriptors, binary fingerprints, molecular graph convolutions) to encode structural characteristics of molecules in machine-readable formats [16]. Over the past two decades, machine learning (ML) and deep learning (DL) approaches have been integrated in QSAR frameworks to improve their predictive accuracy and scalability [17].

Several studies have developed QSARs using both ML and DL strategies to forecast the agonist and antagonist activity of potential PPARγ modulators [18–20]. The combination of molecular descriptors, docking simulations and statistical methods has proven effective in classifying compounds based on their biological activity against PPARγ [21]. Two studies conducted a comprehensive analysis of the NuRA dataset [22], reporting the performance of various classifiers on tasks related to binding, agonism, and antagonism for multiple NRs, including PPARγ [23, 24]. Valsecchi et al. evaluated the performance of single-task classifiers against more computationally demanding multitask deep and shallow neural networks, whilst Ramaprasad et al. used conventional ML algorithms trained on extended connectivity (ECFPs) and Molecular Access System (MACCS) fingerprints. Koh et al. developed a multi-step screening approach that combined multiple ML methodologies used sequentially to identify antagonist candidates and reported a true positive rate of 88% [25]. Moreover, a different study developed two QSARs for PPARγ antagonism with a balanced accuracy score of 83% by external validation and used them to screen 11,092 REACH-registered substances [26].

Recent advances in computational modelling of NR activity mainly relied upon large chemical collections, such as the Tox21 10 K [27] and the NuRA data collections, which contain a diverse array of small molecules from multiple chemical classes. Notably, a significant portion of these datasets consist of per- and polyfluoroalkyl substances (PFAS)-related compounds (i.e., 16.4% of NuRA). PFAS are a group of man-made organofluorine substances, defined as chemicals with at least a perfluorinated methyl (–CF_3_–) or methylene (–CF_2_–) group [28, 29]. Due to their excellent stability, water and strain resistance properties, and surfactant nature, PFAS are commonly used in a variety of industrial and consumer products, such as food packaging, firefighting foams, non-stick cookware, and surface coatings [28, 30, 31]. Despite their useful properties and applications, PFAS are of high concern to environmental regulators since they were found to remain in various environmental compartments for periods of time longer than any other synthetic substance [32]. They were detected in groundwater, surface water, air, and soil [33], demonstrating their pervasive nature and the challenges associated with their remediation. They can also bioaccumulate in the tissues of plants [34] and animals [35], potentially affecting their growth, development, and reproductive health.

The PFAS ability to accumulate within biosystems not only raises ecological concerns but also presents potential risks to human health. Several studies since the 1980s report health outcomes, linking PFAS exposure to immune system dysfunction, increased cancer risk and developmental delays [36]. The toxicity of PFAS and their possible impact on biosystems are being further investigated to comprehend their bioactivity and evaluate them through in vivo and in vitro biological experiments [37]. QSARs have been built by Cheng and Ng [38] to classify the bioactivity of chemical structures from the OECD PFAS list [39], which has since been superseded following updates to the PFAS definition [29], using a panel of 26 biological targets. A significant aspect of this evaluation involves investigating the molecular interactions of PFAS with biomolecules, such as NRs, with recent efforts focussing on elucidating the mechanisms through which PFAS influence PPAR activation [40, 41]. Singam et al. [42] utilised molecular modelling and ML approaches to screen the interactions of a large set PFAS with ten NRs, identifying potential binding patterns.

In the present study, the aim is to develop computational tools for predicting the binding efficiency and antagonistic activity of small molecules targeting PPARγ. A fully connected neural network (NN) was initially trained on ECFPs to estimate binding strength based on molecular docking scores. For antagonist activity, the NN outputs are subsequently used by three base classifiers that represent diverse learning paradigms, which are used in an ensemble bagging strategy to increase accuracy. Given that the QSARs were designed for non-informatics experts (e.g., experimentalists, industry stakeholders, policymakers, and risk assessors), both models were developed according to regulatory guidelines and made available as user-friendly web applications to serve the scientific community. Adhering to core FAIR (Findable, Accessible, Interoperable, and Reusable) principles [43], the data and models are shared through interactive platforms that allow both experts and non-specialists to access and apply them in a reproducible manner. To demonstrate the application of our proposed methodology, we conducted a case study on 34 commercially available PFAS compounds. These prioritised substances were screened using our models to forecast their binding strength and activity with PPARγ.

## Materials and methods

### Data curation

The Tox21 programme is a collaborative initiative aiming to screen a large chemical compound library to assess their biological activity and potential toxicity against a panel of NRs through high-throughput screening (HTS) [27, 44]. For the development of the two predictive models, we used a representative set of the project “qHTS assay to identify small-molecule antagonists of the peroxisome proliferator-activated receptor gamma (PPARγ) signalling pathway: Summary”, associated with the Tox21 programme. In order to identify active PPARγ antagonist compounds, biological activity was determined in vitro, where the Tox21 10 K chemical compound library was tested against the PPARγ-bla HEK293H cell line that contains a beta-lactamase reporter gene. Additional QSARs and modelling studies on PPARγ antagonists, based on this same assay, have been conducted by other researchers [25, 26, 45]. The assay antagonist compounds were sourced from the PubChem Bioassay, under the record AID 743199 (https://pubchem.ncbi.nlm.nih.gov/bioassay/743199) [46]. A total of 6,587 unique compounds were retrieved from the bioassay, after the removal of duplicates, salts, and compounds that consisted of less than 4 atoms. Amongst these, 220 compounds were identified as PFAS, forming a notable subset of the refined dataset.

### Molecular docking calculations

**Fig. 1:**
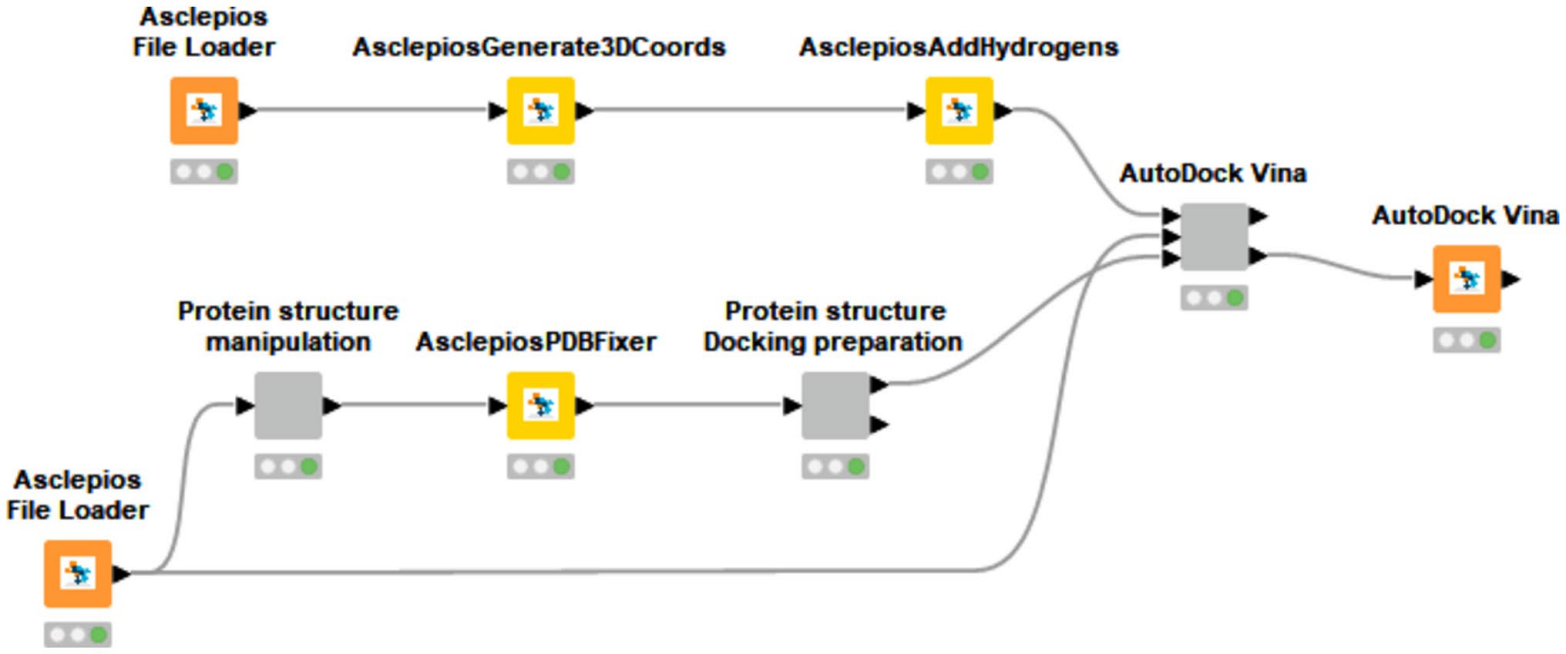
The protein and ligand preparation branches for performing molecular docking calculations with the Enalos Asclepios KNIME pipeline

Our proposed approach involves using the Enalos Asclepios KNIME pipeline [47, 48] (Fig. 1) to perform molecular docking virtual screening on PPARγ-ligand complex model systems for binding affinity predictions. Molecular docking calculations were performed to determine the most favourable binding conformation of the assay compounds considered in the Ligand-Binding Pocket (LBP) of the PPARγ homo sapiens ligand-binding domain structure (PDB IDs: 5YCP) [49]. The removal of heteroatoms, replacement of non-standard residues, and addition of heavy atoms were performed with the AsclepiosPDBFixer node. Ligand missing hydrogen atoms were added via the AsclepiosAddHydrogens node where appropriate, setting the pH value at 7.4, whilst the AsclepiosGenerate3DCooords converted the 2D structure to 3D. Virtual screening calculations were conducted using the Vina-GPU [50, 51] software implementation of the AsclepiosVinaGPU node.

Calculations involved a docking box covering the LBP with *xyz* dimensions set to 25 Å × 25 Å × 25 Å and 1 Å grid spacing. The centre of the grid was placed at the centre of mass coordinates of the crystallographic ligand, namely rosiglitazone. The number of threads was set equal to 8000. After the docking calculations were performed, the compounds whose binding affinities displayed unfavourable interactions (i.e., positive binding energy) were treated as outliers and were omitted from further analysis.

### Deep Learning for binding affinity prediction

#### Data pre-processing

Following the removal of outliers, the remaining dataset contained 6,538 compounds accompanied by a continuous value that represented their binding affinities. Considering the range of binding affinity values (between −13 and −0.6 kcal/mol), a k-means clustering technique was employed to classify the compounds into two distinct categories based on their molecular docking score. The rationale for using unsupervised clustering, rather than a regression setting, lies in the inherent variability of binding affinity values across different scoring functions and computational tools. Instead of selecting a predefined binding energy threshold, which can be arbitrary and may not be universally applicable to all datasets or contexts [52], clustering allows informed separation and a data-driven classification that accounts for the natural distribution of binding affinities. This approach is more practical for virtual screening applications where binary decisions (e.g., binders vs. non-binders) are often more relevant than continuous predictions. Two clusters were created; the former with a cluster centre at -8.06 kcal/mol that corresponds to the class with high binding affinities, whilst the latter with a cluster centre at -5.49 kcal/mol that corresponds to the class with low binding affinities.

Molecular structures were encoded into fixed-length feature vectors using ECFPs, which capture local atomic environments and are widely used for structure–activity modelling. They process molecules of arbitrary size and convert them into fixed-length vectors of 1 s and 0 s that represent the presence or absence of specific features in a molecule [53]. Fingerprints with a radius of 4 and 2048 bits were generated using RDKit and used as input to the NN model. In the next step, data were divided in a random manner into three individual subsets, the training, validation, and test set, following a 70:10:20 ratio. The first set was used to train the NN, the validation set was used for fine-tuning, and the test set was used to evaluate the performance of the final model.

#### Neural network construction

A fully connected NN is a DL architecture composed of layers of interconnected neurons, where each neuron in one layer is connected to every neuron in the subsequent layer [53]. A binary classification model was developed using the DeepChem open-source library (version 2.7.1.), that provides high-quality implementation of molecular featurisation and supports the building of DL architectures for cheminformatics tasks (https://github.com/deepchem/deepchem) [54]. The NN, designed to distinguish between the high and low binding affinity classes, was constructed using the KerasModel class within DeepChem.

Hidden layers used the rectified linear unit (ReLU) activation function, and the final output layer used a sigmoid activation function to produce class probabilities. To determine optimal architecture and training parameters—including the number of neurons in the two hidden layers, the dropout rates for the two dropout layers, the learning rate for the Adam optimiser, and the number of training epochs—a grid search was conducted. The best-performing configuration achieving the highest accuracy based on the validation set was then selected for final training and evaluation. We trained and evaluated two ML models (RF and kNN) to benchmark the NN’s predictive capability against traditional classifiers. The full grid search and optimised hyperparameters of the NN, the RF and kNN models is presented in Table S1 of the Supplementary Information.

### Pipeline for antagonistic activity prediction

A synergistic consensus model was developed for the prediction of PPARγ antagonist activity of small molecules. This approach combines three different modelling methodologies, namely kNN, Random Forest (RF), and Support Vector Machine (SVM), and makes a prediction based on the majority vote of the individual models. The consensus model interfaces with the PPARγ deep learning approach for binding predictions via the Enalos application programming interfaces (APIs), and the categorical labels representing the strength of the interactions (e.g., ‘Strong’/’Weak’ binders) were incorporated as a supplementary attribute in the three individual models. The Isalos Analytics Platform (version 0.2.2), a software that allows straightforward implementation of ML workflows [55], was utilised during the pre-processing process: initial analysis steps, including data preparation and feature selection, were performed using the special functions encoded into the software. Algorithm building and model validation was performed in KNIME Analytics Platform (version 4.7.5) and the ‘Consensus’ Enalos + node obtained the majority vote prediction.

#### Data pre-processing

Firstly, molecular characterisation was generated from the neutralised SMILES notations of the compounds using our in-house ‘EnalosMold2’ node in KNIME, which outputs a diverse set of interpretable molecular descriptors [56]. Mold2 computes 777 one-dimensional (1D) and two-dimensional (2D) structural attributes of each compound, encompassing information on atom counts, bonds, functional groups, physicochemical properties, autocorrelation, charge, connectivity, and topological features. Whilst alternative descriptor sets also offer comprehensive coverage, our comparisons showed that after excluding 3D and non-numerical features, the resulting feature space from Mordred (719 remaining descriptors) was narrower than that of Mold2 with no consistent improvement in model performance. The extracted descriptors were further processed with a low variance filter with an upper bound of 30% that excluded 370 attributes with minimal impact on the target variable.

After compound duplication removal, the produced dataset exhibited a notable imbalance towards the ‘Inactive’ category, with an active rate of ~ 6.5%. Imbalanced datasets, where one class outweighs the other, can introduce bias to a model’s performance, as algorithms tend to favour the majority class resulting in high accuracies but poor detection of the minority class [57]. Previous efforts in QSAR modelling for PPARγ activity used Tomek Link-based under-sampling [58] and others used the Synthetic Minority Over-Sampling technique (SMOTE) to mitigate class imbalance [25, 59]. In this study, the highly skewed category distribution was managed with a random under-sampling (RUS) technique. The number of samples was decreased by randomly removing instances from the majority class and a total of 1230 compounds remained in the dataset to reflect similar-size samples from both classes (410 active and 820 inactive compounds). This ratio was selected as a compromise between managing class imbalance and preserving structural diversity. To further assess the impact of RUS on chemical space representation, the distribution of retained and removed inactives in the chemical space was analysed with t-distributed stochastic neighbour embedding (t-SNE). The resulting plot (Figure S1) shows that both groups are evenly dispersed and demonstrate substantial overlap, suggesting that the chemical diversity of the majority class was preserved during the RUS process, with minimal loss of relevant structural information despite the reduction in sample size.

For feature normalisation, z-score scaling was applied to standardise the independent variables to resemble a Gaussian distribution. To prepare the data for modelling, it was partitioned into three subsets: a training set, a validation set, and a test set split into 70:6:24 proportions with stratified sampling, allowing equal representation of each class to the representative samples.

#### Model development

Following data pre-processing, we considered two different feature selection methods for the identification of the most important variables from the reduced pool of 407 molecular descriptors: (a) The ‘BestFirst’ (forward direction)’ method that performs greedy hill climbing search was used with the CfsSubsetEval wrapper and (b) the ‘InfoGain’ method was used along with the ‘Ranker’ evaluator in order to reduce the dimensionality of the dataset by eliminating redundant features based on their information gain. Supervised classification techniques commonly employed in cheminformatics tasks were trained on the resultant descriptor subsets from each feature selection method, using the Weka extension nodes in KNIME. Three distinct methodologies with dissimilar modelling philosophies were examined: Random Forest (RF) that represent ensemble-based modelling, Support Vector Machines (SVM) as a margin-based classifier, and the k-nearest neighbour (kNN) algorithm that considers distance-based proximity [60].

**Fig. 2:**
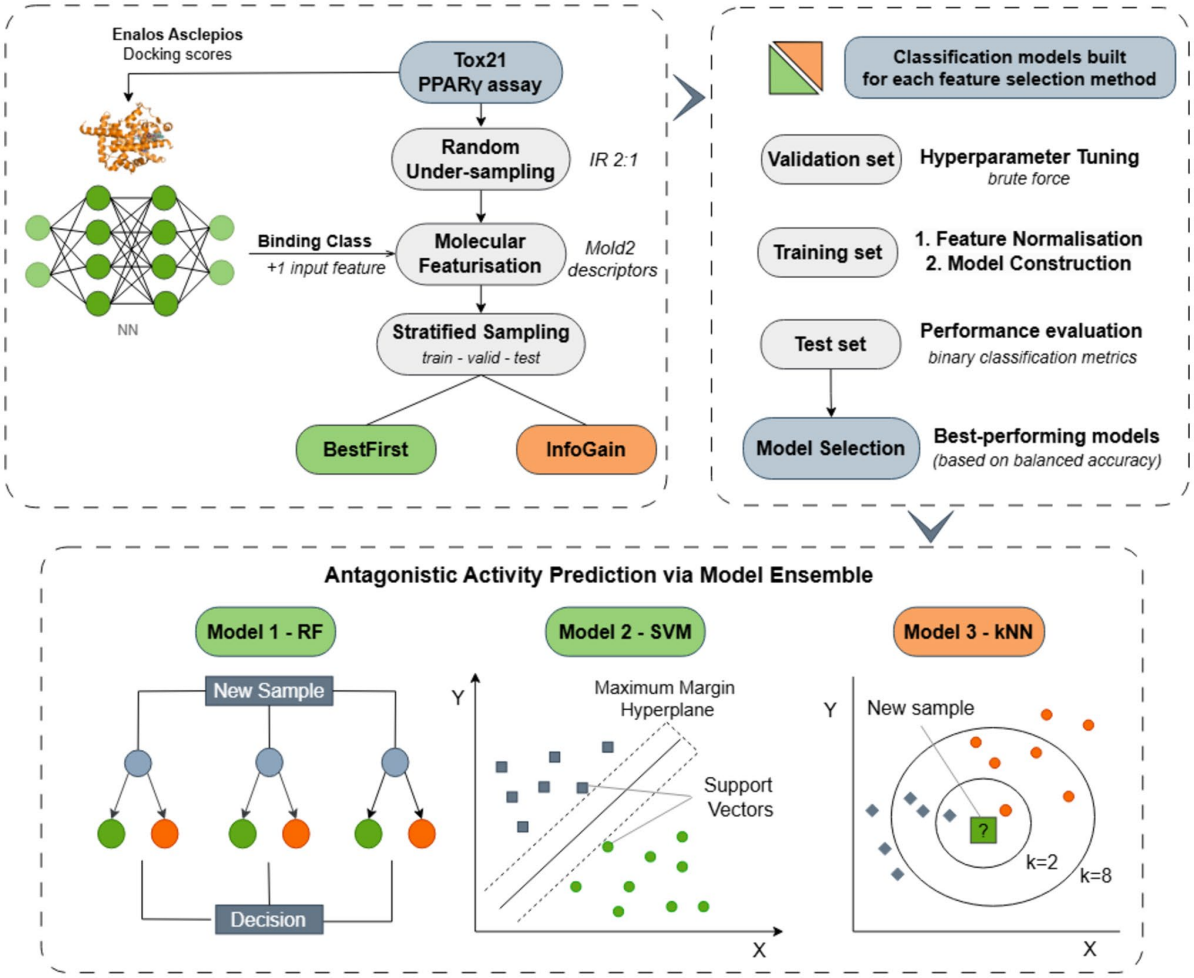
Schematic synopsis of the model development workflow for PPARγ antagonistic activity prediction: Following class balancing into an inactive-to-active ratio (IR) of 2:1 and using Mold2 features for molecular representation, stratified sampling was applied to obtain three subsets. Hyperparameter tuning was conducted on multiple binary classifiers (RF, SVM, kNN) trained on two different feature subsets, and the final consensus is formed by bagging the outputs from the three best-performing models. Model colours in the bottom panel indicate their corresponding feature selection path (green = ’BestFirst’; orange = ’InfoGain’)

Hyperparameter optimisation for each algorithm was conducted with an exhaustive brute-force grid search over a fixed set of values (listed in Table S2), using the validation subset. Each algorithm was looped through all possible combinations of hyperparameters for both descriptor sets, and the configuration yielding the highest balanced accuracy on the validation set was retained. Therefore, the three base classifiers were evaluated for each set of molecular features and the resulting six models were tested on the remaining 24% of the dataset (test set). Amongst these, the top-performing base models (i.e., those with the highest balanced accuracy) were selected to construct a consensus model using a bagging-like voting mechanism [61]. An odd number of models (*n* = 3) were chosen to avoid tied decisions. The overall modelling workflow is presented in Fig. 2.

### Model validation and uncertainty estimation

To assess the predictive capacity of the developed models across the training, validation, and test sets against the categorical endpoints, a range of statistical criteria was utilised. The performance of both models was evaluated based on the number of correct predictions (TP; true positives, TN; true negatives) and the number of misclassifications (FP; false positives, FN; false negatives) [62]. The full list of validation metrics is provided in Table S3 of the Supporting Information for reference. Furthermore, goodness-of-fit and robustness tests were performed through k-fold cross-validation, where the original training set is randomly partitioned into k equal sized subsamples. Of the k subsamples, the remaining k − 1 subsamples are used as the ‘new’ training dataset, whilst a complementary subsample is retained as the validation data for testing the model. Each time, the original training data set is reduced by a smaller group of compounds, thus a new model is developed in every step, and the selection bias that could occur by the selection of only one training set is eliminated [62].

The domain of applicability (APD), defined after model validation, is a key requirement in regulatory QSAR modelling, as it defines the boundaries within which the model’s assumptions can be made with confidence. It provides a measure to distinguish compounds structurally similar to the training instances and those that fall outside a model’s reliable prediction range [63]. In this work, the degree of similarity between a query compound and the training subset was calculated with a distance-based method that considers the Euclidean distances amongst all training samples and compares them to a threshold [64]. For the DL model, pairwise distances amongst the training samples were calculated using the ECFP vectors. The ensemble model utilised the common molecular descriptors used across all the base classifiers, similarly to our previous study [65]. A prediction is deemed unreliable if the computed distance exceeds the predefined APD limit, implying insufficient similarity to compounds represented in the training subset.

## Results and discussion

To build prediction models for PPARγ antagonists, we utilised a refined set of compounds from the Tox21 chemical library, supplemented with molecular docking calculations. The proposed methodology involved constructing a neural network-based model to evaluate the binding efficiency of small molecules with PPARγ and a consensus model to estimate their antagonist activity. The Organisation for Economic Co-operation and Development established principles to guide the scientific community in the development and regulatory approval of QSAR models [66]. These guidelines emphasised a clear definition of endpoints, the use of an unambiguous algorithm, a transparent definition of the model’s applicability domain and appropriate methods to assess goodness-of-fit, robustness, and predictive performance of a model. In addition, a mechanistic interpretation of the selected features was encouraged, where feasible. The need for transparency and reproducibility of QSARs was addressed by the European Commission’s Joint Research Centre, which proposed a QSAR Model Reporting Format (QMRF) template for thorough documentation of model details [67]. In this work, the models were developed in compliance with regulatory standards. This section presents the evaluation of both the NN and ensemble models, describes key molecular descriptors and highlights their dissemination as web applications available for any interested user. The key information of the consensus model was summarised and reported adhering to QMRF guidelines, available in the Supporting Information files accompanying this work (S2).

### Performance evaluation

#### Deep learning model

The performance of the constructed NN was evaluated on the validation and test subsets and benchmarked against two classical ML algorithms under identical training conditions. The fivefold resampling technique was implemented, and each subsample was split into the new holdout and training sets in a ratio of 80:20. Results are reported in Table 1, including average metrics across the five folds. The NN model consistently outperformed both RF and kNN across all evaluation metrics. Notably, it achieved a test accuracy of 0.821, a MCC of 0.628, and a ROC-AUC of 0.893 ± 0.014 on cross-validation. The model indicates consistent performance and generalisation across different folds of the training data. In comparison, the RF and kNN models showed lower test accuracy (0.774 and 0.701, respectively) and weaker MCC values (0.482 and 0.387, respectively). Finally, the APD analysis of the NN model confirmed that the vast majority of test compounds (91.98%) fall within the reliable prediction space, defined by a threshold distance of 7.660.

**Table 1:** Performance comparison of the NN-based model with RF and kNN models, evaluated on validation and test sets

Model	Subset	Accuracy	Sensitivity	F1 Score	Matthews Correlation Coefficient	ROC-AUC score
RF	Validation	0.772	0.907	0.821	0.542	0.865
	Test	0.744	0.911	0.802	0.482	0.863
kNN	Validation	0.701	0.961	0.786	0.412	0.746
	Test	0.694	0.940	0.778	0.387	0.751
NN	Validation	0.846	0.913	0.873	0.681	0.911
	Test	0.821	0.872	0.851	0.628	0.890
	Fivefold CV (holdout set)	0.809 ± 0.010	0.860 ± 0.016	0.838 ± 0.007	0.609 ± 0.020	0.893 ± 0.014

#### Consensus for antagonist activity

For the antagonistic activity against PPARγ, model selection was based on the evaluation of three algorithms trained using two distinct feature selection approaches: BestFirst (forward direction) and Information Gain (InfoGain), applied on the Mold2 descriptor set. The evaluation process examined each model independently and the models achieving the highest balanced accuracy on the test set were used as part of a consensus ensemble under a majority voting scheme. Tables 2 and 3 present the test set and stratified tenfold cross-validation results, respectively.

**Table 2:** Test set validation metrics of the base classifiers using different feature selection methods

	Algorithm	Accuracy	BA	Sn	Pr	Sp	F1 score	Cohen’s kappa
BestFirst	**RF**	**0.834**	**0.803**	**0.714**	**0.769**	**0.893**	**0.741**	**0.619**
	**SGD-SVM**	**0.780**	**0.735**	**0.602**	**0.694**	**0.868**	**0.645**	**0.486**
	kNN	0.769	0.728	0.602	0.670	0.853	0.634	0.467
InfoGain	RF	0.773	0.722	0.571	0.691	0.873	0.626	0.465
	SGD-SVM	0.729	0.659	0.449	0.629	0.868	0.524	0.342
	**kNN**	**0.773**	**0.730**	**0.602**	**0.678**	**0.858**	**0.638**	**0.473**
**Consensus**		**0.858**	**0.835**	**0.765**	**0.798**	**0.904**	**0.781**	**0.676**

*BA Balanced Accuracy, *Sn* Sensitivity, *Pr* Precision, *Sp* Specificity

**Table 3:** Performance metrics of the three individual models and the consensus model evaluated using tenfold-stratified cross-validation. Results are reported as mean ± standard deviation

Statistical measure	RF (Model 1)	SVM (Model 2)	kNN (Model 3)	Consensus
Accuracy	0.821 ± 0.040	0.785 ± 0.039	0.757 ± 0.052	0.813 ± 0.029
Balanced Accuracy	0.781 ± 0.051	0.738 ± 0.049	0.710 ± 0.065	0.769 ± 0.040
Sensitivity	0.662 ± 0.098	0.596 ± 0.098	0.569 ± 0.120	0.638 ± 0.081
Precision	0.771 ± 0.069	0.717 ± 0.072	0.659 ± 0.093	0.764 ± 0.048
Specificity	0.901 ± 0.035	0.880 ± 0.040	0.852 ± 0.051	0.901 ± 0.026
F1 score	0.709 ± 0.074	0.646 ± 0.071	0.605 ± 0.099	0.693 ± 0.056
Cohen’s kappa	0.582 ± 0.099	0.495 ± 0.094	0.433 ± 0.129	0.560 ± 0.073

The final selected models include

- Model 1: A Random Forest model (number of trees = 29), commonly used in QSAR modelling that employs an ensemble learning method, combining the outputs of multiple decision trees to heighten predictive accuracy [68].
- Model 2: Support Vector Machine (learning rate = 0.01, epsilon = 0.001) optimised via Stochastic Gradient Descent with hinge loss, yielding a resilient margin-based classifier that optimises the separation between classes [69].
- Model 3: A k-Nearest Neighbours (k = 8) model, which offers a straightforward non-parametric technique that can be used under a read-across concept, since it allows the exploration of the adjacent chemical space of the compound under study [70].

Models 1 and 2 were developed using the BestFirst feature subset and Model 3 utilised the InfoGain-derived features.

The consensus model displays the highest balanced accuracy and F1 score (83.5% and 78.1%, respectively), and outperforms the distinct models 1–3 across all validation metrics. This suggests that combining the predictions of multiple models through a majority vote results in improved performance compared to using each model individually. For context, a baseline majority class classifier that always predicts the more prevalent class (inactive) yields an overall accuracy of 33.22% and a balanced accuracy of 50%. All tested models significantly outperformed this benchmark. The proposed consensus approach demonstrates comparable or improved predictive performance when compared to previous modelling efforts of the PPARγ antagonist assay. The reliability percentage of the test set was equal to 99.2% (366 out of 369 compounds of the test set), indicating high confidence in the predictions of this subset.

### Interpretation of selected descriptors

According to the principles of OECD, providing a mechanistic interpretation of the key molecular descriptors influencing a model’s predictions is a requisite for its regulatory acceptance [66]. Models 1 (RF) and 2 (SVM) use the same 27 molecular descriptors (Table S4), whilst Model 3 (kNN) employed a different algorithm which identified 24 descriptors (Table S5). The six shared significant molecular descriptors that are commonly used by all three base models are presented in Table 4. The descriptors are ranked based on their information gain scores, which reflect their individual contributions to the base classifier in Model 3.

**Table 4:** List of the 6 common molecular descriptors used across all three models. Descriptor IDs follow the original notations provided by the Mold2 software

Descriptor (Mold2 ID)	Description	Information Gain ranking (Model 3)
D441	Topological structure autocorrelation length 3 weighted by atomic polarizabilities	0.158
D542	Lowest eigenvalue from Burden matrix weighted by van der Waals order 3	0.149
D545	Lowest eigenvalue from Burden matrix weighted by van der Waals order 6	0.144
D574	Highest eigenvalue from Burden matrix weighted by van der Waals order 3	0.174
D575	Highest eigenvalue from Burden matrix weighted by van der Waals order 4	0.161
D576	Highest eigenvalue from Burden matrix weighted by van der Waals order 5	0.162

Firstly, the Broto-Moreau spatial autocorrelation descriptor (ATSp,3) was commonly identified as the significant descriptor for all three ML algorithms. Molecules with higher polarizability distribution are more likely to exhibit PPARγ biological activity. This measure, introduced in the 1980s, evaluates the distribution of atomic properties within a molecule’s topology [71, 72], here weighted by atomic polarizability (Eq. S1). This molecular descriptor captures how an atomic property is distributed throughout the molecular structure [73]. This family of autocorrelation descriptors has been used in previous studies to estimate the biological activity and toxicity of small molecules [74–76]. Burden eigenvalues also emerged as critical descriptors in all three individual models. This descriptor is a chemically intuitive molecular index computed as a solution to the characteristic equation of the Burden matrix, an H-depleted modified connectivity matrix (Eq. S2). Here, the diagonal elements of matrix *B* are determined by the normalised van der Waals volume values [73].

These findings are consistent with previous studies on PPARγ modulators, where indices such as Burden eigenvalues and centred Moreau-Broto autocorrelation descriptors were also identified as the most influential non-3D descriptors during model construction [18]. This agreement further supports the relevance of these features in capturing key topochemical and electronic properties associated with PPARγ activity.

### Model deployment and data accessibility

**Fig. 3:**
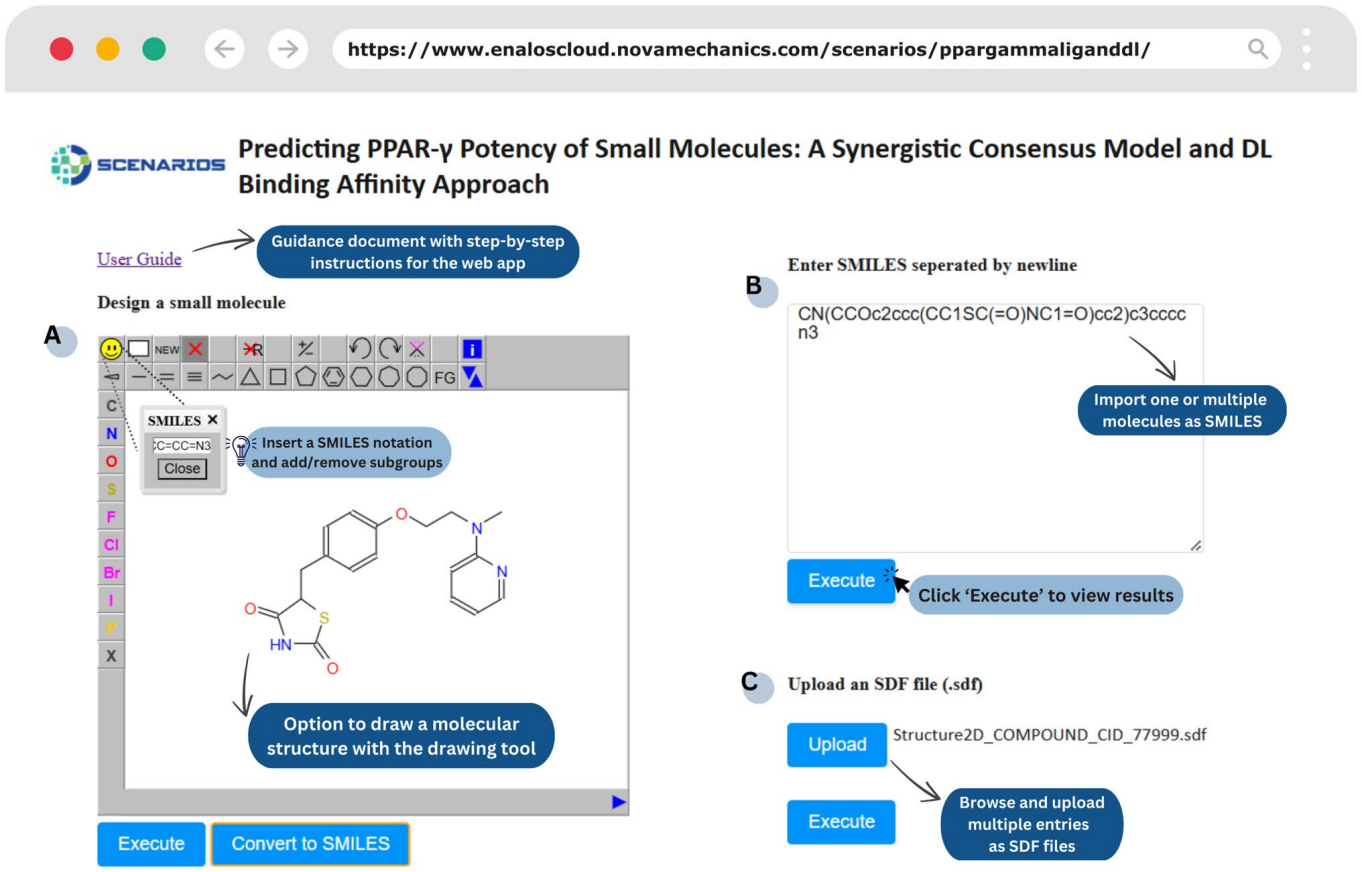
The PPARγ Consensus model environments in the Enalos Cloud Platform: **A** the Design Molecule, **B** the SMILES, and **C** the SDF fields for input compounds

In an attempt to facilitate the assessment of small molecules against the PPARγ nuclear receptor, the DL approach for binding affinity prediction, as well as the consensus model for PPARγ antagonist potency were integrated as web applications via the Enalos Cloud Platform. The Enalos Cloud Platform hosts ready-to-use web applications of cheminformatics [65, 77–79] and materials informatics [70, 80, 81] predictive models for accelerated drug discovery, biological activity estimation, and nanomaterials risk assessment. These in silico models are freely offered as Graphical User Interfaces (GUIs) designed for non-informatics researchers, reducing the number of experimental assays and minimising associated costs. Both user interfaces allow small-molecule submission in different formats by sketching a molecule and converting it to SMILES code or by uploading an SDF file containing a large number of small molecules (Fig. 3). The outputs are offered in machine-readable structures (e.g., CSV files). The web tools developed for the two workflows can be easily accessed through the links: https://www.enaloscloud.novamechanics.com/scenarios/ppargamma/ and https://www.enaloscloud.novamechanics.com/scenarios/ppargammaliganddl/.

NR-ToxPred, a web server developed by Ramaprasad et al., allows the classification of small molecules based on their binding properties to multiple NRs, including PPARγ [23]. Whilst NR-ToxPred is capable of broader screening against nuclear receptors for both agonist and antagonist tasks, its design requires user-defined settings for the definition of APD space and applies multiple classifiers, which can lead to different outputs depending on input criteria. In comparison with the previously established tool, our implementation is focussed on delivering rapid, reproducible outputs with uncertainty estimations specifically for antagonist activity.

A Representational State Transfer (REST) API was developed to improve the accessibility of the two predictive models and made available online via the following link: https://enaloscloud.novamechanics.com/scenarios/swagger-ui/index.html). The API employs the POST Request Method, chosen for its ability to securely handle and transfer large volumes of structured input data. The use of an API for model availability enables seamless integration of the developed workflows into diverse systems and platforms, allowing users to utilise the model’s capabilities without requiring direct access to the computational setup. Users can submit input data, such as single or multiple SMILES strings representing chemical compounds of interest, using a JSON-formatted tuple and interact with the PPARγ antagonist bioactivity prediction models. The API interface offers user-friendly endpoints for PPARγ predictions, supporting SMILES and SDF inputs via POST requests.

**Fig. 4:**
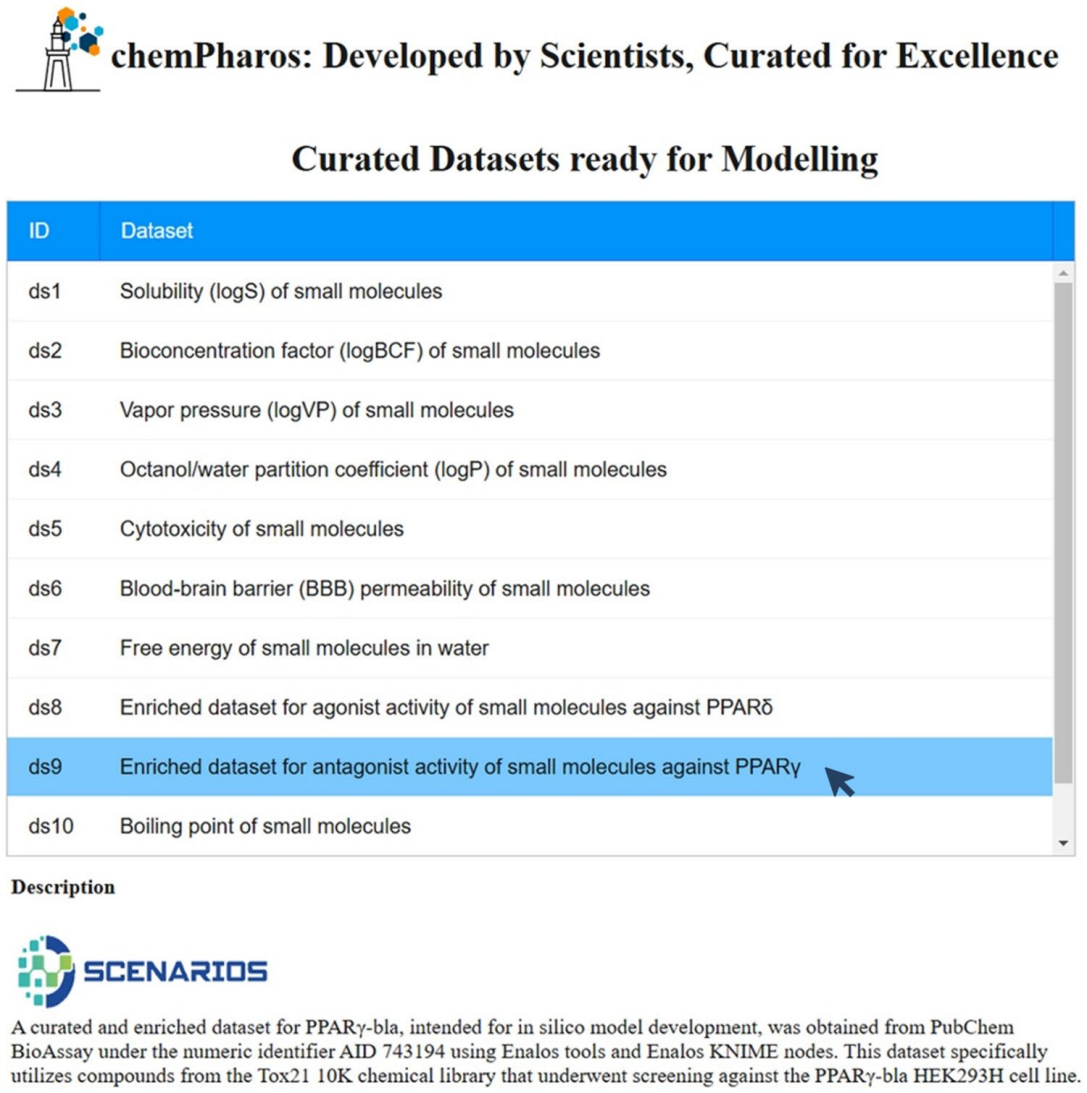
The ChemPharos interface of curated datasets, including the augmented dataset ‘ds9’ used in this study, designed for analysing and modelling chemical properties

All information, including the raw and enriched data with calculated molecular descriptors and docking calculations used in this study, are publicly available in a QSAR-ready format and can be accessed via ChemPharos at https://db.chempharos.eu/datasets/Datasets.zul?datasetID=ds9. The ChemPharos database (Fig. 4) stores ready-to-use datasets for the rapid modelling of small molecules’ chemical properties and functionalities. This study is largely aligned with the FAIRification framework by openly distributing the training datasets and the developed predictive models and by providing programmatic access via APIs [82].

### Targeting PFAS for PPARγ binding and activity prediction

Owing to the impact of PFAS over the NRs, we conducted a case study with PFAS commonly utilised in the industry to demonstrate the utility of our proposed methodology. Over 7 million PFAS-related chemicals, as defined by the refined OECD PFAS criteria, are catalogued in PubChem [29, 83]. Comprehensible experimental testing of such a vast number of compounds for their bioactivity and interactions with PPARγ is impractical. To address this issue, we selected 34 commercially available PFAS compounds from diverse subfamilies (which did not appear in the final training sets), including perfluoroalkyl carboxylic acids and sulfonic acids (PFCAs/PFSAs), perfluoroalkyl ether carboxylic acids, and sulfonic acids (PFECAs/PFESAs) for evaluation using our computational pipelines.

Despite being classified as “Strong” or “Weak” binders based on docking scores, none of the selected PFAS compounds exhibited antagonist activity (Table 5). The predicted probabilities from the NN model corresponding to higher binding affinities are also reported to denote the confidence of each classification. All but one of the 34 compounds included in this evaluation were assessed within the applicability domain of the NN and consensus models. A Bemis-Murcko scaffold analysis was conducted to understand the chemical diversity within the PFAS subset [84]. The subset has a lower ratio of unique scaffolds (number of scaffolds (N)/number of compounds (M) = 51.36%) compared to the full dataset (N/M = 29.46%), but the fragment of singleton scaffolds (Ns) is similar (Ns/N = 80.53% for the subset and Ns/N = 75.49% for the full set). This implies that despite its small size, the PFAS subset is structurally diverse and exhibits similar degree of chemical diversity as the broader dataset. Nevertheless, PFAS compounds remain underrepresented in the training set relative to the full chemical library, which may impact model generalisability and prediction confidence.

**Table 5:** Binding affinity and antagonist activity predictions for 34 PFAS compounds using the developed in silico models

a/a	PubChem CID	Commercial Name	Binding Class	Confidence	Antagonist activity
1	15099039	8:2 Cl-PFESA	Strong Binder	0.910	Inactive
2	114481	GenX	Weak Binder	0.755	Inactive
3	87119578	–	Weak Binder	0.964	Inactive
4	61890	Nafion By-product 1	Weak Binder	0.658	Inactive
5	12498036	PFMOBA	Weak Binder	0.012	Inactive
6	120228	PFMOPrA	Weak Binder	0.988	Inactive
7	75922	PFPeS	Strong Binder	0.999	Inactive
8	67820	PFHpS	Strong Binder	0.999	Inactive
9	23671509	–	Weak Binder	0.627	Inactive
10	14317645	Nafion By-product 2	Strong Binder	0.988	Inactive
11	4604312	–	Strong Binder	0.657	Inactive
12	2776108	PFEESA	Weak Binder	0.988	Inactive
13	2769352	–	Strong Binder	0.999	Inactive
14	2760333	PFO3TrDA	Strong Binder	0.999	Inactive
15	2778260	PFO3DoDA	Weak Binder	0.997	Inactive
16	2778677	–	Weak Binder	0.502	Inactive
17	2782393	PFO2HpA	Weak Binder	0.999	Inactive
18	182328	HFPO-TeA	Strong Binder	0.676	Inactive
19	93076	HFPO-TA	Weak Binder	0.784	Inactive
20	2782557	Branched ADONA	Strong Binder	0.883	Inactive
21	5125273	PFMOAA	Weak Binder	0.995	Inactive
22	23678874	PFHxS-K	Strong Binder	0.999	Inactive
23	23669238	PFOS-K	Strong Binder	0.999	Inactive
24	67821	PFNA	Strong Binder	0.999	Inactive
25	2776282	PFOA-NH4	Weak Binder	0.837	Inactive
26	77222	PFUnA	Strong Binder	0.998	Inactive
27	9554	PFOA	Strong Binder	0.995	Inactive
28	67815	PFBS	Strong Binder	0.878	Inactive
29	62356	PFPrA	Weak Binder	0.988	Inactive
30	9555	PFDA	Strong Binder	0.999	Inactive
31	75921	PFPeA	Strong Binder	0.994	Inactive
32	67818	PFHpA	Strong Binder	0.998	Inactive
33	67542	PFHxA	Strong Binder	0.993	Inactive
34	9777	PFBA	Strong Binder*	0.848	Inactive

*Compounds falling outside the APD of the NN model

In line with the findings of Table 5, a recent study by Singam et al. [42] also predicted inactivity for commercially significant PFAS in terms of antagonist activity. Specifically, although 9 of the selected PFAS did not appear in their dataset, the remaining compounds from our collection were classified as ‘Inactive’ regardless of displaying varying binding affinity scores. However, structure–function relationships in NRs cannot be explained by binding energetics alone. For example, it has been reported in literature that in NRs like the retinoid X receptor alpha (RXRα), the oestrogen receptor alpha (ERα), and PPARα, depending on the presence of agonist, antagonist, or no ligand, the commonly shared H12 adopts distinct conformations [85–87]. In PPARγ, agonists stabilise H12 in its active conformation through key hydrogen bonds, most notably with Tyr473 [88], thereby ligand-driven conformational changes collectively control its activation. Studies on PFAS alternatives, such as ADONA and GenX which are included in our list, have shown that whilst these compounds exhibit significantly favourable binding energies with PPARγ, molecular dynamics simulations revealed that not all formed hydrogen bonds with Tyr473 [89]. Even though our model demonstrates consistent predictions for the selected PFAS compounds regarding PPARγ, molecular docking calculations are inherently limited in making a definitive prediction of the binding pose or determining whether this pose is related to interactions responsible for receptor activation or inhibition [90]. Although no antagonist activity amongst the tested PFAS is predicted, this does not preclude the possibility of agonistic or partial agonistic effects. Structural studies have shown that some PFAS, such as PFOA, bind to PPARγ LBD and may recruit coactivators or stabilise active conformations, acting as partial agonists [91]. Molecular dynamics simulations also indicate that PFAS can interact with multiple sites on PPARγ, potentially influencing receptor activity through non-classical pathways [89]. These findings underscore that strong binding is not exclusive to antagonists and highlight the complexity of PFAS-mediated PPARγ modulation.

Additionally, the Ligand-Binding Domains (LBDs) of the three isoforms of the PPAR nuclear receptor subfamily retain the fold characteristics typical of nuclear receptors’ LBDs, comprised 13 α-helices and a small four-stranded β-sheet, and share considerable amino acid identity (ranging from 54% to 71% in humans) [92–94]. Despite the conserved amino acid sequences amongst these subtypes, our model was solely trained on the bioassay scores and docking calculations against the PPARγ receptor. Depending on data availability, the proposed methodology can be modified and expanded for other NRs. Bearing these considerations in mind underscores the utility of our models in evaluating the potential bioactivity of these compounds against PPARγ.

Detailed screening for eight physicochemical and biological properties of the 34 PFAS compounds—water solubility, vapour pressure, boiling point, octanol/water partition coefficient, bioconcentration factor, mutagenicity, experimental hydration free energy in water, and blood–brain barrier permeability—was implemented through the Titania web suite [95]. The calculation of these properties offers complementary information into the environmental and biological behaviour of the selected PFAS. Property estimations, along with a reliability indication of each prediction for all tested compounds, are provided as Supporting Information.

## Conclusion

The present work investigates the interactions of a large ensemble of small molecules with the PPARγ biomolecule using in silico tools. A fully connected NN model was developed to categorise small molecules into two distinct classes based on their molecular docking scores. The DL model, trained on fingerprint vectors, outputs a classification of a query compound as either a ‘Strong’ or ‘Weak’ binder, reflecting high or low binding affinity, respectively. A subsequent model employs molecular descriptors extracted from the molecules’ 2D structures and communicates with the NN via Enalos APIs, to incorporate information of the molecules’ binding interactions to PPARγ. Three independent algorithms relying on ensemble learning, gradient descent, and read-across methodologies were combined into a unified approach that outputs the majority vote for antagonist activity prediction of compounds against PPARγ. The consensus model achieved an overall balanced accuracy of 83.5%, outperforming the three base classifiers in their ability to detect the minority class (Model 1: 80.3%, Model 2: 73.5%, Model 3: 73.0%).

The two models were validated in accordance with regulatory guidelines using external validation and k-fold cross-validation tests. The domain of applicability was assessed to guarantee the reliability of predictions generated for novel compounds. In line with OECD principles, we provided a mechanistic interpretation by identifying key molecular descriptors, such as polarizability-weighted autocorrelation indices and Burden eigenvalues that are associated with the topochemical and electronic properties driving PPARγ antagonist activity. Adhering to regulatory requirements, both models can be accessed through freely available, user-friendly GUIs on the Enalos Cloud Platform, ensuring broad usability, particularly for users without programming expertise. These web applications can be used by researchers and stakeholders as screening tools to explore PPARγ interactions and accelerate the discovery of novel modulators.

In addition, a case study using commercially relevant PFAS was conducted to demonstrate the practical applicability of our models, where 34 selected compounds were classified as inactive in terms of PPARγ antagonistic activity. Despite the limited representation of the PFAS chemical class (*n* = 220) on our initial dataset, fragment analysis revealed adequate structural diversity, with over 80% of scaffolds being unique to a single compound. Whilst docking scores quantify binding affinity, they do not inherently predict whether a compound will act as antagonist or not. These observations emphasise the need for experimental validation of computational predictions, as well as the need to expand chemical libraries to include a broader range of structurally diverse PFAS screened against nuclear receptors.

## Ethical approval

Not applicable.

## Supplementary Information

Below is the link to the electronic supplementary material.

Supplementary file1 (DOCX 8049 KB)

## Data Availability

The curated dataset and its accompanying enrichment attributes are openly accessible through ChemPharos, via the following link: https://db.chempharos.eu/datasets/Datasets.zul?datasetID=ds9.

## Abbreviations

APD: Applicability domain threshold
API: Application Programming Interfaces
DL: Deep Learning
ECFP: Extended Connectivity Fingerprints
ERα: Oestrogen receptor alpha
FAIR: Findable-Accessible-Interoperable-Re-usable
FN: False negative
FP: False positive
GUI: Graphical User Interface
H12: Helix 12
HTS: High-throughput screening
LBD: Ligand-Binding Domain
LBP: Ligand-Binding Pocket
NR: Nuclear Receptor
OECD: Organisation for Economic Co-operation and Development
PFAS: Per- and polyfluoroalkyl substances
PFCA/PFSA: Perfluoroalkyl Carboxylic Acids/Sulfonic Acids
PFECA/PFESA: Perfluoroalkyl Ether Carboxylic Acids/Sulfonic Acids
PPAR: Peroxisome proliferator-activated receptor
PPRE: Peroxisome proliferator response element
QMRF: QSAR model reporting format
QSAR: Quantitative structure–activity relationship
ReLU: Rectified Linear Unit
REST: Representational State Transfer
RF: Random Forest
ROC-AUC: Receiver Operating Characteristic – Area under the Curve
RXR: Retinoid X Receptor
SDF: Structure data file
SMILES: Simplified Molecular Input Line Entry System
SVM: Support Vector Machine
t-SNE: T-distributed stochastic neighbour embedding
TN: True negative
TP: True positive
kNN: K-Nearest neighbours

## Code availability

The Enalos Asclepios KNIME nodes and Isalos Analytics Platform (https://isalos.novamechanics.com/) workflow used in this study are proprietary to NovaMechanics Ltd. and require a licensing agreement for access.
