# Supplementary material for: Predicting peroxisome proliferator-activated receptor gamma potency of small molecules: a synergistic consensus model and deep learning binding affinity approach powered by Enalos Cloud Platform
Source: Mol Divers. 2025 Jun 14;30(2):1821–36. doi: 10.1007/s11030-025-11230-6 (PMC13139292; doi:10.1007/s11030-025-11230-6)
Supplement: Supplementary file 1 — Supplementary file1 (DOCX 8049 KB) [file 11030_2025_11230_MOESM1_ESM.docx]

**Supporting Information**

Predicting Peroxisome Proliferator-Activated Receptor gamma Potency of Small Molecules: A Synergistic Consensus Model and Deep Learning Binding Affinity Approach powered by Enalos Cloud Platform

Maria Antoniou^1,2,3^, Konstantinos Papavasileiou^3,4^, Antreas Tsoumanis^1,3,4^, Georgia Melagraki^5^, Antreas Afantitis^1,3,4*^

^1^Department of ChemoInformatics, NovaMechanics Ltd, Nicosia, 1070, Cyprus.

^2^Computation-Based Science and Technology Research Centre, The Cyprus Institute, Nicosia, 2121, Cyprus.

^3^Entelos Institute, Larnaca, 6059, State, Cyprus.

^4^Department of ChemoInformatics, NovaMechanics MIKE, Piraeus, 18545, Greece.

^5^Division of Physical Sciences Applications, Hellenic Military Academy, Vari, 16672, Greece.

*Corresponding author(s). E-mail(s): [afantitis@novamechanics.com;](mailto:afantitis@novamechanics.com)

**
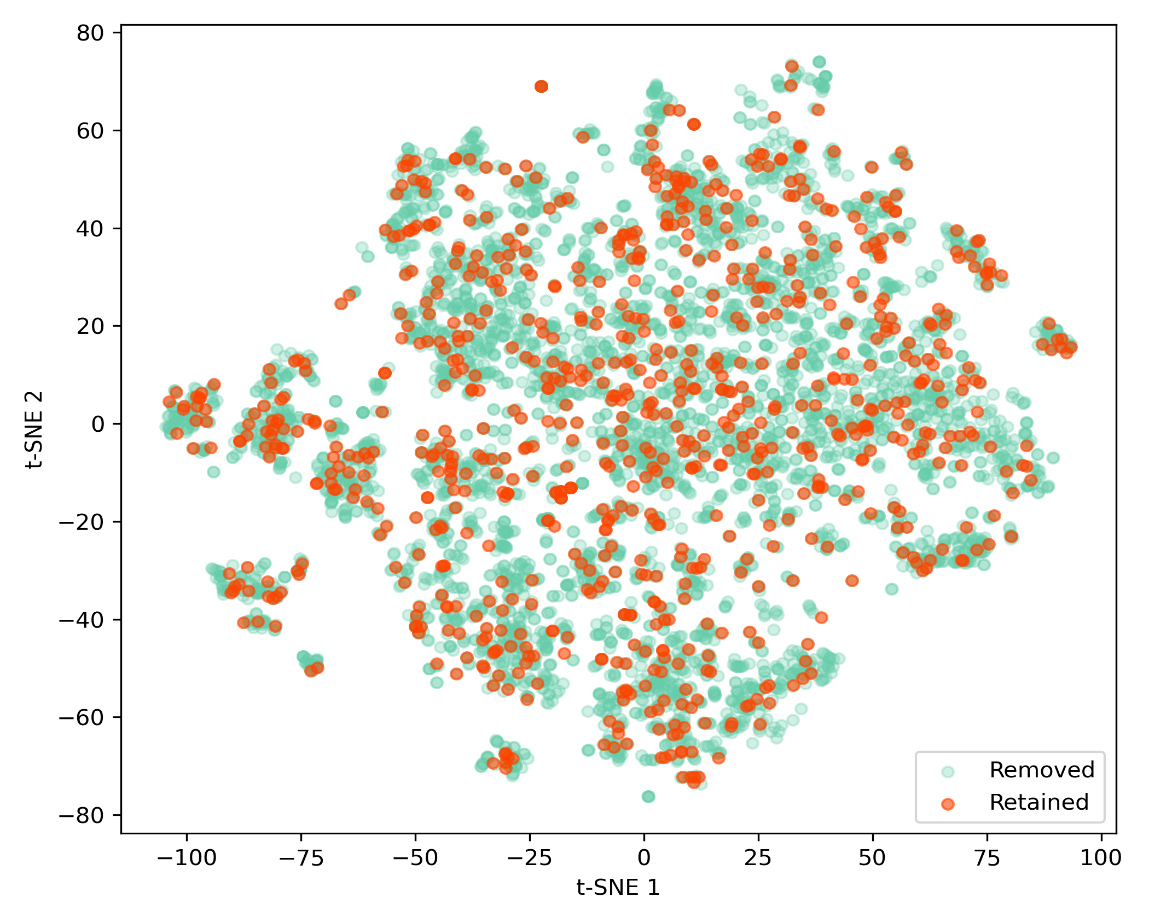
Figure S1**: t-SNE distribution of the molecular descriptor space after under-sampling the majority class. Selected (orange) and excluded (green) inactive compounds are visualised based on the full set of Mold2 descriptors.


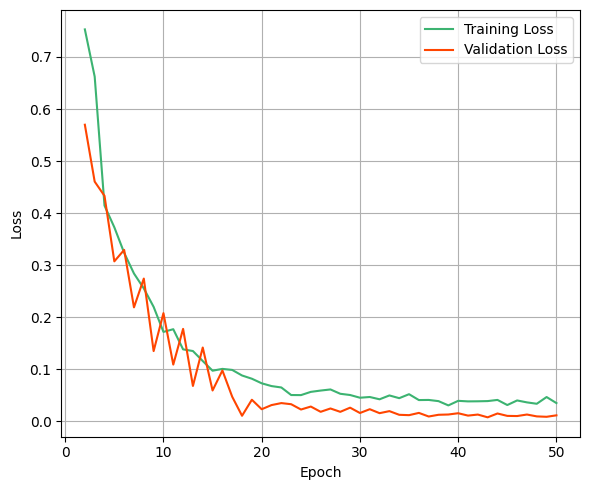


**Figure S2:** Training and validation loss per epoch during the training of the binary classification model over 50 epochs.

**Table S1:** Hyperparameter grids and optimal values for the neural network and RF classifiers trained on ECFPs for binding affinity prediction.

| Algorithm | Tested hyperparameter grids | Optimal value |
| --- | --- | --- |
| RF | Number of Estimators ∈ {10, 20, 30, 40} | 20 |
|  | Maximum Depth ∈ {1, 2, 5, 10} | 10 |
| kNN | Number of Neighbours ∈ {3, 4, …, 9, 10} | 4 |
|  | Weight Function ∈ {uniform, distance} | distance |
| NN | Number of units in the first Dense Layer  ∈ {32, 64, 128} | 32 |
|  | Dropout rate after Dense Layer 1  ∈ {0.2, 0.3, 0.4, 0.5} | 0.2 |
|  | Number of units in second Dense Layer  ∈ {16, 32, 64} | 64 |
|  | Dropout rate after Dense Layer 2 ∈ {0.3, 0.4, 0.5, 0.6} | 0.6 |
|  | Learning Rate ∈ {0.001, 0.0005} | 0.001 |
|  | Number of Epochs ∈ {5, 25, 50} | 25 |

**Table S2:** Hyperparameter grids and optimal configurations per feature selection strategy for the six base classifiers used in predicting antagonistic activity towards PPARγ.

| Algorithm | Tested hyperparameter grids | Optimal values - Mold2 | |
| --- | --- | --- | --- |
|  |  | BestFirst | InfoGain |
| RF | Number of Estimators ∈ {5, 6 , …, 34, 35} | 29 | 25 |
|  | Maximum Depth ∈ {0, 1, 2, 3} | 0 | 0 |
| SVM | Number of Iterations ∈ {10, 15, 20, 25} | 20 | 30 |
|  | Learning Rate ∈ {0.01, 0.05, 0.1, 0.5} | 0.01 | 0.05 |
| kNN | Number of Neighbours ∈ {3, 4, …, 9, 10} | 4 | 8 |
|  | Weight Function ∈ {uniform, distance} | distance | distance |

**Table S3.** Statistical measures used to evaluate the classification models

| Metric | Metric Formula |
| --- | --- |
| Accuracy | $\frac{TP+TN}{TP+TN+FP+FN}$ |
| Balanced Accuracy | $\frac{1}{2} \left( \frac{TP}{TP+FN}+ \frac{TN}{TN+FP} \right)$ |
| Sensitivity | $\frac{TP}{TP+FN}$ |
| Precision | $\frac{TP}{TP+FP}$ |
| Specificity | $\frac{TN}{TN+FP}$ |
| F1-Score | $\frac{2TP}{2TP+FP+FN}$ |
| Matthews Correlation Coefficient | $\frac{TP\times TN-FP\times FN}{\sqrt{(TP+FP)(TP+FN)(TN+FP)(TN+FN)}}$ |
| Cohen’s kappa | $\frac{2(TP\times TN-FP\times FN)}{\left( TP+FP \right)\left( FP+TN \right)+(TP+FN)(TN+FN)}$ |

TP: True Positive (correctly predicted actives); TN: True Negative (correctly predicted inactives); FP: False Positive (incorrectly predicted as active); FN: False Negative (incorrectly predicted as inactive)

**Table S4.** Mold2 molecular descriptors selected with ‘BestFirst’, used for the development of Model 1 (Random Forest) and Model 2 (Support Vector Machines).

| Descriptor ID | Mold2 - Descriptor Name |
| --- | --- |
| D186 | Average vertex distance connectivity index |
| D255 | Vertex distance count equality index |
| D272 | Information content order-3 index |
| D277 | Total information content order-2 index |
| D282 | Structural information content order-1 index |
| D374 | Sum of topological distance between the vertices O and O |
| D384 | Sum of topological distance between the vertices S and Cl |
| D396 | Sum of topological distance between the vertices Cl and Cl |
| D397 | Sum of topological distance between the vertices Cl and Br |
| D419 | Topological structure autocorrelation length-5 weighted by atomic masses |
| D441 | Topological structure autocorrelation length-3 weighted by atomic polarizabilities |
| D503 | Moran topological structure autocorrelation length-1 weighted by atomic polarizabilities |
| D505 | Moran topological structure autocorrelation length-3 weighted by atomic polarizabilities |
| D541 | Lowest eigenvalue from Burden matrix weighted by van der Waals order-2 |
| D542 | Lowest eigenvalue from Burden matrix weighted by van der Waals order-3 |
| D545 | Lowest eigenvalue from Burden matrix weighted by van der Waals order-6 |
| D574 | Highest eigenvalue from Burden matrix weighted by van der Waals order-3 |
| D575 | Highest eigenvalue from Burden matrix weighted by van der Waals order-4 |
| D576 | Highest eigenvalue from Burden matrix weighted by van der Waals order-5 |
| D588 | Highest eigenvalue from Burden matrix weighted by polarizabilities order-1 |
| D595 | Highest eigenvalue from Burden matrix weighted by polarizabilities order-8 |
| D599 | Number of total quaternary C-sp3 |
| D604 | Number of substituted aromatic C-sp2 |
| D738 | Number of group R~CR~R |
| D746 | Number of group H attached to C0(sp3) no X attached to next C |
| D754 | Number of group O= |
| D777 | Molecular regression coefficients surface LogP index |

**Table S5:** Mold2 molecular descriptors selected with ‘InfoGain’, used for the development of Model 3 (k-Nearest Neighbours).

| Descriptor ID | Mold2 Description |
| --- | --- |
| D024 | Number of Carbon |
| D143 | Sum of atomic Van Der Waals Carbon-Scele |
| D151 | Sum of atomic polarizabilities scaled on Carbon-SP3 |
| D212 | Valence vertex connectivity order-0 Index |
| D424 | Topological structure autocorrelation length-2 weighted by atomic van der Waals volumes |
| D429 | Topological structure autocorrelation length-7 weighted by atomic van der Waals volumes |
| D439 | Topological structure autocorrelation length-1 weighted by atomic polarizabilities |
| D440 | Topological structure autocorrelation length-2 weighted by atomic polarizabilities |
| D441 | Topological structure autocorrelation length-3 weighted by atomic polarizabilities |
| D442 | Topological structure autocorrelation length-4 weighted by atomic polarizabilities |
| D443 | Topological structure autocorrelation length-5 weighted by atomic polarizabilities |
| D542 | Lowest eigenvalue from Burden matrix weighted by van der Waals order-3 |
| D543 | Lowest eigenvalue from Burden matrix weighted by van der Waals order-4 |
| D544 | Lowest eigenvalue from Burden matrix weighted by van der Waals order-5 |
| D545 | Lowest eigenvalue from Burden matrix weighted by van der Waals order-6 |
| D546 | Lowest eigenvalue from Burden matrix weighted by van der Waals order-7 |
| D547 | Lowest eigenvalue from Burden matrix weighted by van der Waals order-8 |
| D574 | Highest eigenvalue from Burden matrix weighted by van der Waals order-3 |
| D575 | Highest eigenvalue from Burden matrix weighted by van der Waals order-4 |
| D576 | Highest eigenvalue from Burden matrix weighted by van der Waals order-5 |
| D577 | Highest eigenvalue from Burden matrix weighted by van der Waals order-6 |
| D578 | Highest eigenvalue from Burden matrix weighted by van der Waals order-7 |
| D579 | Highest eigenvalue from Burden matrix weighted by van der Waals order-8 |
| D592 | Highest eigenvalue from Burden matrix weighted by polarizabilities order-5 |

**S1. Definitions of Molecular Descriptors**

- Broto-Moreau Autocorrelation of Topological Structure (ATS) descriptors: They capture weighted correlations between an atomic property at a fixed topological distance. A representative example ATS_p3_ is defined as:

$$\begin{aligned} {ATS}_{p3}=\frac{1}{2}\cdot\sum_{i=1} \sum_{j=1} w_{i}\cdot w_{j}\cdot\delta\left( d_{ij};m \right)\#\left( Eq. S1 \right) \end{aligned}$$

where, *δ* is the Kronecker delta function that equals to 1 if the topological distance *d_ij_=m=3*, zero otherwise and *w_i,j_* are the normalised atomic polarizability of atoms *i* and *j*, respectively. The topological distance *d_ij_* measures the number of involved bonds along the shortest path between two atoms.

- Burden Matrix Eigenvalues: The n^th^ highest or lowest eigenvalues are determined by solving the general eigenvalue equation:

$$\begin{aligned} B\cdot V=V\cdot e \#\left( Eq. S2 \right) \end{aligned}$$

where, *V* is a matrix of eigenvectors, and *e* is a diagonal matrix of eigenvalues whose elements correspond to a topological distance between pairs of atoms.

**S2.** Dissemination of the consensus model using the QMRF reporting template

| **1.** | **QSAR identifier** | |
| --- | --- | --- |
| 1.1. | QSAR identifier (title) | Development of synergistic consensus model for the cytotoxicity prediction of PPARγ antagonists |
| 1.2 | Other related models | Deep Learning model for the binding affinity prediction of PPARγ antagonists, integrated into the consensus model |
| 1.3. | Software coding the model | Isalos Analytics Platform (<https://isalos.novamechanics.com/>) & KNIME Analytics Platform (v.4.7.5.)  The model was implemented as a web application, available at:  <https://www.enaloscloud.novamechanics.com/scenarios/ppargammaliganddl/> |
| **2.** | **General information** | |
| 2.0 | Abstract | The consensus predictive model utilises 1230 compounds from the Tox21 10K chemical library that have been screened against the PPARγ-bla HEK293H cell line, which contains a beta-lactamase reporter gene. The original bioassay includes the molecules’ cytotoxicity measured through cell viability tests and a classification as 'active'/'inactive' antagonists, based on their AC50 (potency) score. The predictions from a deep learning model categorising the compounds as ‘Strong’ or ‘Weak’ binders to the PPARγ receptor. The model employs a consensus model consisting of three machine learning algorithms and adheres to the guidelines established by the OECD. |
| 2.1. | Date of QMRF | - |
| 2.2. | QMRF author(s) and contact details | Maria Antoniou – [antoniou@novamechanics.com](mailto:antoniou@novamechanics.com)  Antreas Afantitis – afantitis@novamechanics.com |
| 2.3. | Date of QMRF update(s) | Not Applicable |
| 2.4. | QMRF update(s) | Not Applicable |
| 2.5. | Model developer(s) and contact details | Maria Antoniou – [antoniou@novamechanics.com](mailto:antoniou@novamechanics.com)  Konstantinos Papavasileiou – [papavasileiou@novamechanics.com](mailto:papavasileiou@novamechanics.com)  Antreas Tsoumanis - [tsoumanis@novamechanics.com](mailto:tsoumanis@novamechanics.com)  Georgia Melagraki - [georgiamelagraki@gmail.com](mailto:georgiamelagraki@gmail.com)  Antreas Afantitis - [afantitis@novamechanics.com](mailto:afantitis@novamechanics.com) |
| 2.6. | Date of model development and/or publication | Date of publication: May 2025 |
| 2.7. | Reference(s) to main scientific papers and/or software package | Predicting PPARγ Potency of Small Molecules: A Synergistic Consensus Model and Deep Learning Binding Affinity Approach |
| 2.8. | Availability of information about the model | The model is non-proprietary: full description of the model algorithm is available; training and test sets are available. The model is implemented in a public web service and the full dataset with data enrichment attributes is publicly available at the following link: <https://db.chempharos.eu/datasets/Datasets.zul?datasetID=ds9> |
| 2.9. | Availability of another QMRF for exactly the same model | Not Applicable |
| **3** | **Defining the endpoint - OECD Principle 1: “A DEFINED ENDPOINT"** | |
| 3.1. | Species | Small Molecules |
| 3.2. | Endpoint | Prediction of the potency class (‘Active’ or ‘Inactive’) of small molecules to peroxisome proliferator-activated receptor gamma (PPARγ) |
| 3.3 | Comment on endpoint | Classification in two categories is determined from the relative activity scores of the initial compounds, which are obtained based on the logarithmically transformed half-maximal efficacy (AC_50_) values. |
| 3.4. | Endpoint units | None |
| 3.5. | Dependent variable | Two potency categories, ‘Active’ and ‘Inactive’. |
| 3.6. | Experimental protocol | The assay compounds of PubChem Bioassay record AID 743194 (https://pubchem.ncbi.nlm.nih.gov/bioassay/743194) were used for the development of the predictive model. This bioassay is a representative set of the project "qHTS assay to identify small molecule antagonists of the peroxisome proliferator-activated receptor gamma (PPARg) signaling pathway: Summary", associated with Tox21 programme. The Tox21 10K chemical compound library was tested against the PPARγ-bla HEK293H cell line that contains a beta-lactamase reporter gene. A total of 6587 compounds were retrieved from the PubChem bioassay, after the removal of duplicates, salts, and compounds that consisted of less than 4 atoms.  More information on the experimental protocol can be found in: <https://pubchem.ncbi.nlm.nih.gov/bioassay/743194> |
| 3.7. | Endpoint data quality and variability | A total of 267 compounds which were assigned in the ‘Inconclusive’ potency class (PUBCHEM_ACTIVITY_SCORE between 1-39) were excluded from the dataset, as well. The remaining dataset was highly imbalanced since the ‘Active’ class comprised only 6.5% of the total compounds (410 out of 6320). |
| **4** | **Defining the algorithm - OECD Principle 2 : “AN UNAMBIGUOUS ALGORITHM”** | |
| 4.1. | Type of model | Consensus model considering the majority votes from three base models: a non-parametric supervised learning algorithm (k-NN), an ensemble learning algorithm (Random Forest) and a discriminative equation-based model (SVM) that optimises class separation. |
| 4.2. | Explicit algorithm | - Model 1: Random Forest (RF) algorithm: Employs multiple decision trees (number of trees = 29) - Model 2: Binary Support Vector Machine (SVM): A margin-based classifier that utilises stochastic gradient descent for hinge loss minimisation (epochs = 20, learning rate = 0.01, epsilon = 0.001) - Model 3: k-Nearest Neighbours (k-NN) algorithm (k = 8): This algorithm identifies the k-number of training data points that are ‘closest’ to the unclassified instance in the test set. Distances are assigned based on Euclidean distances, and inverted distance is used as a weighting factor for the k nearest points.   A consensus model is built based on the majority vote from the predictions provided by each of the individual models. |
| 4.3. | Descriptors in the model | Molecular descriptors refer to descriptors based on the one-dimensional and two-dimensional structure of a compound. As described in Section 4.4., models 1 (RF) and 2 (SVM) employ the same feature selection technique, thus the two models use the same 27 descriptors. Model 3 employed a different algorithm which selected 24 descriptors. Details of each group of molecular descriptors can be found in the Supporting Information files of the original publication. The six shared significant molecular descriptors that are commonly employed by all three models are presented here:   1. Topological structure autocorrelation length-3 weighted by atomic polarizabilities [D441] 2. Lowest eigenvalue from Burden matrix weighted by van der Waals order-3 [D542] 3. Lowest eigenvalue from Burden matrix weighted by van der Waals order-6 [D545] 4. Highest eigenvalue from Burden matrix weighted by van der Waals order-3 [D574] 5. Highest eigenvalue from Burden matrix weighted by van der Waals order-4 [D575] 6. Highest eigenvalue from Burden matrix weighted by van der Waals order-5 [D576] |
| 4.4. | Descriptor selection | Feature selection was performed to select the most important variables from an initial pool of 777 molecular descriptors. After applying a low-variance filter to filter out descriptors that have less impact on the target variable, 407 attributes remained.  The ‘BestFirst (forward direction)' approach was utilised for the identification of the most important descriptors used by the SVM and RF algorithms. The ‘BestFirst’ approach uses a greedy algorithm that iteratively adds or removes features to find the successor in all the tested combinations. descriptors were selected, along with binding affinity calculations, a descriptor that was manually selected.  The ‘InfoGain’ method, employed by the k-NN algorithm, was used along with the ‘Ranker’ evaluator in order to reduce the dimensionality of the dataset by eliminating irrelevant features based on their information gain. The ‘Ranker’ technique prioritises the variables and removes the lower-ranking ones, improving the predictive performance of the k-NN algorithm. |
| 4.5. | Algorithm and descriptor generation | All of the 777 molecular descriptors were generated using the 'EnalosMold2' KNIME node that employs the Mold2.exe executable and an SDF file to calculate molecular descriptors. |
| 4.6. | Software name and version for descriptor generation | Mold2 is a software developed by the National Centre for Toxicological Research (NCTR) that calculates a large and diverse set of 777 molecular descriptors encoding two-dimensional chemical structure information. More information on the Mold2 software can be found in <https://www.fda.gov/science-research/bioinformatics-tools/mold2> |
| 4.7. | Chemicals/Descriptors ratio | 1230 chemicals: 27 descriptors (Models 1&2) / 24 descriptors (Model 3)  A random under-sampling technique was used to select representative samples from the ‘Inactive’ class, resulting in an inactive-to-active ratio of 2:1. |
| **5** | **Defining the applicability domain - OECD Principle 3: “A DEFINED DOMAIN OF APPLICABILITY”** | |
| 5.1. | Description of the applicability domain of the model | The applicability domain is defined by fixed boundaries, the APD threshold, calculated by considering Euclidean distances between all molecules in the training set. If the domain of a molecule is beyond this threshold, then the prediction is considered unreliable.  More information on the applicability domain methodology can be found in:   - G. Melagraki, Α. Afantitis, H. Sarimveis, P.A. Koutentis, O. Igglessi – Markopoulou, G. Kollias "In Silico Exploration for Identifying Structure–Activity Relationship of MEK Inhibition and Oral Bioavailability for Isothiazole Derivatives" Chemical Biology and Drug Design 2010; 76: 397–406, <https://doi.org/10.1111/j.1747-0285.2010.01029.x> - Melagraki G, Ntougkos E, Rinotas V, Papaneophytou C, Leonis G, et al. (2017) Cheminformatics-aided discovery of small-molecule Protein-Protein Interaction (PPI) dual inhibitors of Tumor Necrosis Factor (TNF) and Receptor Activator of NF-κB Ligand (RANKL). PLOS Computational Biology 13(4): e1005372. <https://doi.org/10.1371/journal.pcbi.1005372> |
| 5.2. | Method used to assess the applicability domain | The domain of applicability of the consensus model is calculated using the six molecular descriptors shared by all three involved algorithms. The distance of a test molecule to its nearest neighbour in the training set in compared to the pre-defined APD threshold, APD='d'+'stdev'*z.   - First, the average Euclidean distances between all pairs of training data is calculated and then the set of distances that were lower than the average is formulated. - The 'd' and 'stdev' values are finally determined as the average and standard deviation of all distances included in the remaining set. - z is an empirical parameter with a value of 0.5 |
| 5.3. | Software name and version for applicability domain assessment | ‘Domain-APD’ node from the Enalos+ extension for the KNIME Analytics Platform (version 4.7.5) |
| 5.4. | Limits of applicability | APD threshold = 1.255, If the domain of a molecule is lower than this threshold, then the prediction is considered reliable. The reliability percentage of the testing set equals 99.2% (366 out of 369 compounds of the test set). |
| **6** | **Defining goodness-of-fit and robustness (internal validation) – OECD Principle 4: “APPROPRIATE MEASURES OF GOODNESS-OF-FIT, ROBUSTENESS AND PREDICTIVITY”** | |
| 6.1. | Availability of the training set | It is available but not attached. The whole set of the filtered molecular descriptors can be accessed via ChemPharos (<https://db.chempharos.eu/datasets/Datasets.zul?datasetID=ds9>). |
| 6.2. | Available information for the training set | Available information for the training set:  a) SMILES notations  b) InChiKeys codes  c) PubChem Compound ID and ChEMBL ID. |
| 6.3. | Data for each descriptor variable for the training set | The descriptor values of the training set are available via ChemPharos. |
| 6.4. | Data for the dependent variable for the training set | The dependent variable values of the training set are available via ChemPharos. |
| 6.5. | Other information about the training set | The initial dataset was partitioned into three representative subsets, the training, test and blank set. The training set percentage was set at 70%, thus it consists of 861 small molecules selected for model development. Also, the training set includes 574 molecules characterised as ‘Inactive’, and 287 molecules characterised as ‘Active’. |
| 6.6. | Pre-processing of data before modelling | - Low Variance filter: Cutoff limit of 20% to filter out columns. - Removal of duplicate molecules - Under-sampling technique: To limit the imbalance presented in the initial dataset. - Z-score normalization: Gaussian distribution. - Feature selection was performed as described in Section 4.4. |
| 6.7. | Statistics for goodness-of-fit | Performance metrics based on test set: Confusion matrix, Accuracy, Balanced Accuracy, Sensitivity, Precision, F1-score, Cohen’s kappa |
| 6.8. | Robustness - Statistics obtained by leave-one-out cross-validation | Not Applicable |
| 6.9. | Robustness - Statistics obtained by leave-many-out cross-validation | Stratified 10-Fold Cross Validation  Accuracy = 0.812 ± 0.045,  Balanced Accuracy = 0.769 ± 0.065,  Sensitivity = 0.638 ± 0.129,  Precision = 0.757 ± 0.064,  Specificity = 0.899 ± 0.025,  F1 Score = 0.688 ± 0.092,  Cohen’s kappa = 0.556 ± 0.118 |
| 6.10. | Robustness - Statistics obtained by Y-scrambling | Not Applicable |
| 6.11. | Robustness - Statistics obtained by bootstrap | Not Applicable |
| 6.12. | Robustness - Statistics obtained by other methods | -Statistics using a blank subset that was kept for hyperparameter tuning consisted of 74 small molecules (6% of the initial dataset):  Accuracy = 0.851,  Sensitivity = 0.760,  Precision = 0.792,  F1-score = 0.776,  Cohen’s kappa = 0.664  - Comparison with majority class predictor:  Baseline accuracy = $\frac{179}{295}$ = 60.7%  Baseline balanced accuracy = 50% |
| **7** | **Defining predictivity (external validation) – OECD Principle 4: “APPROPRIATE MEASURES OF GOODNESS-OF-FIT, ROBUSTENESS AND PREDICTIVITY”** | |
| 7.1. | Availability of the external validation set | It is available but not attached. More information including the data enhancement attributes of the test set can be accessed via ChemPharos (<https://db.chempharos.eu/datasets/Datasets.zul?datasetID=ds9>). |
| 7.2. | Available information for the external validation set | Available information for the test set:  a) SMILES notations  b) InChiKeys codes  c) PubChem Compound ID and ChEMBL ID. |
| 7.3. | Data for each descriptor variable for the external validation set | The descriptor values of the test set are available via ChemPharos. |
| 7.4. | Data for the dependent variable for the external validation set | The dependent variable values of the test set are available via ChemPharos. |
| 7.5. | Other information about the external validation set | 295 (197 inactives, 98 actives) out of the 1230 initial compounds were included in the test set. The test set was not involved in model development, but it was used solely for validating purposes. |
| 7.6. | Experimental design of test set | The initial dataset was split into a 70:30 ratio with stratified sampling for the separation into two representative subsets. The smaller subset was divided with random partitioning into an 80:20 ratio, to obtain the test and blank set respectively. |
| 7.7. | Predictivity - Statistics obtained by external validation | True Positives = 75, False Positives = 19, True Negatives = 178, False Negatives = 23,  Accuracy = 0.858,  Balanced Accuracy = 0.835,  Sensitivity = 0.765,  Precision = 0.798,  F1-score = 0.781,  Specificity = 0.904,  Cohen’s kappa = 0.676 |
| 7.8. | Predictivity - Assessment of the external validation set | The external validation set is sufficiently large and representative of the original data, after applying a random under-sampling technique in the ‘Inactive’ category. It was further assessed using the applicability domain limits. The external validation subset (test set) is 24% of the initial dataset, and 99.2% the instances of the test sets fall within the domain of applicability. |
| 7.9. | Comments on the external validation of the model | Z-score normalization of the test and blank sets was applied based on the calculations on the training set. |
| **8** | **Providing a mechanistic interpretation - OECD Principle 5: “A MECHANISTIC INTERPRETATION, IF POSSIBLE”** | |
| 8.1. | Mechanistic basis of the model | The Broto-Moreau descriptor is a measure whereby the atoms of a molecule are represented by an atomic property, here weighted by atomic polarizabilities. Topological distances are the number edges along the shortest path between two specific atoms, measuring the number of involved bonds. This molecular descriptor provides information on how the atomic property is distributed on the topological structure of the molecule, thus higher polarizability distribution within the molecule contributes to the biological activity of PPARγ. The Burden eigenvalues descriptor is a chemically intuitive molecular index computed as a solution to the characteristic equation of the Burden matrix, an H-depleted modified connectivity matrix. The n^st^ highest or lowest eigenvalues are determined by solving the general eigenvalue equation of a diagonal matrix whose elements correspond to a topological distance between pairs of atoms. Here the diagonal elements of matrix are given by the normalised van der Waals volume values. |
| 8.2. | A priori or a posteriori mechanistic interpretation | A posteriori mechanistic interpretation: the selected descriptors’ possible effect on the end point was analysed after model development. |
| 8.3. | Other information about the mechanistic interpretation | Not Applicable |
| **9** | **Miscellaneous information** |  |
| 9.1. | Comments | Not Applicable |
| 9.2. | Bibliography | • AID 743194 - qHTS assay to identify small molecule antagonists of the peroxisome proliferator-activated receptor gamma (PPARg) signaling pathway - cell viability counter screen - PubChem. <https://pubchem.ncbi.nlm.nih.gov/bioassay/743194>  • Huang, R.; Xia, M.; Cho, M.-H.; Sakamuru, S.; Shinn, P.; Houck, K. A.; Dix, D. J.; Judson, R. S.; Witt, K. L.; Kavlock, R. J.; Tice, R. R.; Austin, C. P. Chemical Genomics Profiling of Environmental Chemical Modulation of Human Nuclear Receptors. Environ Health Perspect 2011, 119 (8), 1142–1148. https://doi.org/10.1289/ehp.1002952.  • Mold(2), molecular descriptors from 2D structures for chemoinformatics and toxicoinformatics (Hong et al., 2008) DOI: 10.1021/ci800038f  • NovaMechanics Ltd, “Enalos + KNIME nodes,” 2017. <http://enalosplus.novamechanics.com/>  • Melagraki G, Ntougkos E, Rinotas V, Papaneophytou C, Leonis G, et al. (2017) Cheminformatics-aided discovery of small-molecule Protein-Protein Interaction (PPI) dual inhibitors of Tumor Necrosis Factor (TNF) and Receptor Activator of NF-κB Ligand (RANKL). PLOS Computational Biology 13(4): e1005372. <https://doi.org/10.1371/journal.pcbi.1005372> |
| 9.3 |  | Not Applicable |
